# Expression of cellular protective proteins SIRT1, HSP70 and SOD2 correlates with age and is significantly higher in NK cells of the oldest seniors

**DOI:** 10.1186/s12979-017-0085-4

**Published:** 2017-01-23

**Authors:** Lucyna Kaszubowska, Jerzy Foerster, Jan Jacek Kaczor, Daria Schetz, Tomasz Jerzy Ślebioda, Zbigniew Kmieć

**Affiliations:** 1 Department of Histology, Medical University of Gdańsk, Dębinki 1, Gdańsk, PL-80-211 Poland; 2 Department of Social and Clinical Gerontology, Medical University of Gdańsk, Dębinki 1, Gdańsk, PL-80-211 Poland; 3 Department of Physiotherapy, Gdansk University of Physical Education and Sport, Górskiego 1, Gdańsk, PL-80-336 Poland; 4 Department of Clinical Toxicology, Medical University of Gdańsk, Kartuska 4/6, Gdańsk, PL-80-104 Poland

**Keywords:** NK cells, Ageing, Immunosenescence, Seniors, SIRT1, HSP70, SOD2, Oxidative stress, Innate immunity

## Abstract

**Background:**

NK cells are key effector lymphocytes of innate immunity provided with constitutive cytolytic activity, however, their role in human ageing is not entirely understood. The study aimed to analyze the expression of proteins involved in cellular stress response sirtuin 1 (SIRT1), heat shock protein 70 (HSP70) and manganese superoxide dismutase (SOD2) in non-stimulated NK cells of the oldest seniors (*n* = 25; aged over 85; mean age 88 years) and compare with NK cells of the old (*n* = 30; aged under 85; mean age 76 years) and the young (*n* = 32; mean age 21 years) to find potential relationships between the level of expression of these proteins in NK cells and longevity. The concentration of carbonyl groups and 8-isoprostanes in NK cell lysates reflecting the level of oxidative stress was also measured.

**Results:**

The group of the oldest seniors differed from the other age groups by significantly higher percentage of NK cells expressing SIRT1, HSP70 and SOD2. The concentration of both carbonyl groups and 8-isoprostanes in NK cell extracts remained within the normal range in all age groups. The percentage of NK cells with the expression of, respectively, SIRT1, HSP70 and SOD2 correlated positively with age. Some correlations between expression levels of particular protective proteins SIRT1, HSP70 and SOD2 were observed in the study population.

**Conclusions:**

The increased expression of cellular protective proteins SIRT1, HSP70 and SOD2 in NK cells of the oldest seniors seems to correspond to longevity and the observed correlations may suggest the involvement of these proteins in establishing NK cell homeostasis specific for healthy ageing process.

## Background

The main function of NK cells is related to immune response against viral infections, tumor cells and intracellular pathogens. These lymphocytes reveal also some regulatory properties to activate other cells of both innate and adaptive immunity by secretion of cytokines and chemokines [20]. NK cells do not act in a constant manner but rather adapt to the temporary conditions of the cellular environment. They can display a form of antigen-specific immunologic memory demonstrating attributes of both innate and adaptive immunity [52]. Human NK cells consist of two functional subsets differing in the cell surface expression of CD56, i.e. immunoregulatory CD56^bright^ cells, considered to be precursors to the cytotoxic NK CD56^dim^ cells [18, 44]. These NK cell subsets are differentially affected by the process of ageing, i.e. more immature CD56^bright^ cells are decreased whereas more differentiated CD56^dim^ cells are expanded [18, 20, 31].

Ageing is associated with the up-regulation of the inflammatory responses caused by chronic antigenic stress and dysregulation of cytokine secretion with increased serum levels of pro-inflammatory cytokines IL-6 and TNF, increased level of C reactive protein and decreased concentration of anti-inflammatory cytokine IL-10 [8]. This phenomenon was referred to as “inflamm-aging” [17]. The ageing process is also accompanied by chronic oxidative stress that affects all regulatory body systems, including the immune system, and this phenomenon has been called “oxi-inflamm-aging” [14]. Oxidative stress can cause serious cell damage of cells, counteracted by the development of anti-oxidant protective systems that involve glutathione (GSH), glutathione S-transferase, glutathione peroxidase, glutathione reductase, catalase, and superoxide dismutases (MnSOD, CuZnSOD) [25]. Continuous low-dose oxidative stress during ageing results in adaptive responses based on the activation of NF-κB and subsequently superoxide dismutases (SODs) with a key role played by the mitochondrial manganese dismutase (SOD2). In this process also heat-shock proteins (HSP70) are induced to protect cells from stress-induced molecular damage [9]. HSP70 reveals distinct functions depending on its location. Intracellular HSP70 presents a cytoprotective, anti-apoptotic and anti-inflammatory function while extracellular HSP70 mediates pro-inflammatory immunological response via Toll-like receptors (TLRs) contributing to a link between innate and adaptive immune systems [3].

Sirtuins are evolutionary conserved proteins with deacetylase activity dependent on nicotinamide adenine nucleotide (NAD+) considered as histone deacetylases involved in the control of ageing and longevity, DNA repair, transcriptional silencing, apoptosis and cellular metabolism [47]. SIRT1 acetylates forkhead transcription factor class O (FOXO) upon peroxide stress and that results in the activation of SOD2 and subsequent reduction of the level of reactive oxygen species [21].

There are no literature data concerning changes in expression of SIRT1, SOD2 and HSP70 in NK cells in the process of ageing. Generally, a decline in function of chaperones in aged organisms was reported [2, 30, 40]. This tendency concerned also basal expression of HSP72 in human lymphocytes and monocytes or induced expression after heat shock [55]. Higher basal levels of HSP70 and several other families of HSP proteins in non-stimulated monocytes and lymphocytes of seniors suggested the maintenance of HSP system at activation state in the elderly [41]. Moreover, it was shown that elevated expression of HSPs was associated with longevity and their decreased levels corresponded to increased protein deterioration during ageing, loss of protein quality control, degeneration and cell death [11].

HSP proteins are expressed also on the cell surface and circulate in peripheral blood. PBMCs (Peripheral Blood Mononuclear Cells) are thought to be a main source of these circulating proteins [41]. The previous studies showed an apparent decrease in serum levels of HSP60 and HSP70 during ageing [45]. Increased serum level of these proteins corresponded to inflammation process and frailty in seniors [42]. However, these data refer to HSP proteins released into plasma in response to a cellular stress by different types of PBMCs.

SIRT1 has been shown to play a major role in the determination of lifespan and stress resistance [6, 9, 49]. In humans levels of SIRT1, SIRT2 and SIRT3 were analyzed in sera of both frail and non-frail seniors and the lower circulating SIRT1 and SIRT3 levels were found to be a distinguishing marker of frailty [29].

SOD2 similarly to SIRT1 seems to play a role in longevity [46]. Experiments on mice with heterozygous deficiency of manganese superoxide dismutase (SOD2) demonstrated that SOD2 was involved in the regulation of “inflamm-aging” of the aged skin immune system. The impairment of SOD2 and reduction of its antioxidative capacities promoted proinflammatory effects through alteration of dendritic cells and T cell functions [50].

Despite the established role of HSP70, SIRT1 and SOD2 proteins in the ageing process [22, 39, 54], little is known about their contribution to healthy ageing of the immune system, and NK cells in particular. There are only few studies concerning this issue and they refer to monocytes [41], lymphocytes [23, 41] and granulocytes [28]. There was observed age-related raise in the basal level of HSP70 in monocytes and lymphocytes [41]. Previous study, however, showed a significant age-related decrease in HSP70 levels in lymphocytes [23]. Studies performed on neutrophils did not expose any significant age-dependent changes in HSP70 basal content in the cells [28]. Since NK cells are associated with the process of healthy ageing, the preservation of NK cell function until very advanced age may contribute to longevity [12, 18, 31]. Therefore, the aim of our study was to analyze the expression of cellular protective proteins in NK cells of the oldest seniors (over 85 years old), the old (aged under 85) and the young subjects (aged 19–24 years).

## Methods

### Participants

Eighty-seven volunteers aged between 19 and 94 years (63 women and 24 men) participated in this study. The exclusion criteria included: CRP > 5 mg/L, cancer, autoimmune disease, diabetes, infection, use of immunosuppressors, glucocorticoids or non-steroid anti-inflammatory drugs (NSAIDs); moderate to severe dementia assessed using the “Mini Mental State Examination” (MMSE below 23 points) [16]. Senior volunteers were also considered with geriatric conditions. Katz’s scale was used to assess “Activities of Daily Living” (ADL) and only seniors with 5–6 points were enrolled to the study [26]. Senior volunteers were recruited among inhabitants of local senior houses and young volunteers were students of Medical University of Gdańsk, Poland. The participants were subdivided into 3 groups including: 32 young subjects referred to as ‘young’ (mean age 21.0 ± 0.3 years, range 19–24 years, 23 women and 9 men); 30 seniors aged under 85 referred to as ‘old’ (mean age 75.6 ± 0.9 years, range 65–84 years, 20 women and 10 men) and 25 seniors at the age over 85 referred to as the ‘oldest’ (mean age 88.4 ± 0.5 years, range 85–94 years; 20 women and 5 men). All volunteers signed informed consent and the study received approval from Ethical Committee of Medical University of Gdańsk, Poland (225/2010).

### Separation of peripheral blood mononuclear cells

Peripheral blood mononuclear cells (PBMCs) were isolated from venous blood samples collected in tubes with EDTA by conventional Ficoll-Uropoline density gradient centrifugation. PBMCs were then washed and resuspended in PBS-1% FBS solution.

### Staining of surface and intracellular antigens for flow cytometry

Whole blood samples (0.1 ml) were aliquoted into flow cytometry tubes and CD3-FITC-conjugated (0.125 μg/ml; clone UCHT1) (BD Biosciences, San Jose, CA, USA) or CD3-PE-Cy7-conjugated (0.125 μg/ml; clone SK7) (BD Biosciences, San Jose, CA, USA), CD56-APC-conjugated (0.6 μg/ml; clone NCAM16.2) (BD Biosciences, San Jose, CA, USA) and Hsp70-PE-conjugated (1 μg/ml; clone N27F34) (Abcam, Cambridge, England) monoclonal antibodies were added for cell surface antigen staining. After 30 min of incubation in the dark at room temperature 2 ml of BD FACS Lysing Solution was added and samples were incubated for subsequent 10 min in the same conditions. Then cells were washed twice with 1 ml of BD Staining Buffer (PBS without Ca^2+^ and Mg^2+^, 1% FBS, 0.09% sodium azide) and resuspended in 0.25 ml of Fixation/Permeabilization Solution for 20 min at 4 °C according to manufacturer’s protocol (BD Cytofix/Cytoperm Fixation/Permeabilization Kit). Cells were washed twice with 1 ml of BD Perm/Wash buffer and appropriate volumes of MnSOD-FITC-conjugated (1 μg/ml; clone MnS-1) (eBioscience, San Diego, CA, USA), Hsp70-PE-conjugated (1 μg/ml; clone N27F34) (Abcam, Cambridge, England), SIRT1-Alexa Fluor 488 – conjugated (1 μg/ml; clone 19A7AB4) (Abcam, Cambridge, England), TNF-PE-Cy7- conjugated (0.125 μg/ml; clone MAb11) (BD Biosciences, San Jose, CA, USA) and IFN-γ-PE-conjugated (0.125 μg/ml; clone 4S.B3) (BD Biosciences, San Jose, CA, USA) monoclonal antibodies were added for staining of intracellular antigens according to the manufacturer’s instructions. After 30 min of incubation in the dark at room temperature cells were washed twice with 1 ml of BD Perm/Wash buffer and resuspeded in Staining Buffer prior to flow cytometric analysis. Samples were run on a BD FACSCalibur flow cytometer equipped with argon-ion laser (488 nm) and data were analyzed with BD CellQuest Pro software (BD Biosciences, San Jose, CA, USA) after acquiring 10,000 gated events (lymphocytes). NK cells were identified as CD3-CD56+ cells. Appropriate isotype controls for both surface and intracellular staining were also prepared. Staining and fixation procedure were carried out within 4 h after blood sample collection.

### NK cell separation for measurement of protein carbonyl groups content and 8-isoprostanes level in cell lysates

NK cells (CD3^−^CD56^+^) were isolated from PBMCs by negative selection with the use of Human NK Cell Enrichment Kit and EasySep Magnet (Stemcell Technologies, Vancouver, Canada). PBMCs were incubated with EasySep Human NK Cell Enrichment Cocktail (a suspension of monoclonal antibodies bound in bispecific Tetrameric Antibody Complexes (TAC) directed against cell surface antigens on human blood cells: CD3, CD4, CD14, CD19, CD20, CD36, CD66b, CD123, HLA-DR, glycophorin A and dextran for 10 min, then vortexed for 30 s and incubated with EasySep D Magnetic Particles (a suspension of magnetic dextran iron particles) for subsequent 5 min. Then cells were resuspended in 2.5 ml of recommended medium (PBS with 2% FBS and 1 mM EDTA, Ca^2+^ and Mg^2+^ free), mixed gently and placed into the magnet. After 2.5 min the desired fraction was poured off into a new tube. Aliquots of the cell fractions were stained with appropriate volumes of CD56-PE- and CD3-PerCP-conjugated monoclonal antibodies (BD Biosciences, San Jose, CA, USA). After 30 min of incubation in the dark at room temperature cells were washed with 2 ml of BD CellWASH solution and finally 0.5 ml of BD CellFIX solution was added. Samples were stored at 4 °C up to 24 h until analyzed by flow cytometry to check the purity of the enriched NK cell fractions and all presented almost 95% purity.

Then, NK cell extracts were prepared with the use of Mammalian Cell & Tissue Extraction Kit (BioVision Research Products, Mountain View, CA, USA) according to the manufacturer’s protocol. The total protein concentration of samples was determined with Bradford assay (Sigma - Aldrich, Saint Louis, MO, USA). Cell lysates were stored at -70 °C for further analysis. The content of protein carbonyl groups in NK cell extracts was measured with the BioCell PC Test Kit, an enzyme-linked immunosorbent assay (BioCell, Auckland, New Zealand). Samples were prepared for ELISA procedure according to the manufacturer’s protocol. Absorbances were measured at 450 nm with Bio-Rad plate reader. A standard curve reflecting absorbances of the increasing concentrations of protein carbonyls in the supplied oxidized protein standards was prepared and used to measure the content of carbonyl groups in experimental samples. Data in the study are presented as nanomoles of carbonyl groups per mg of cellular extract protein (nmol/mg).

The concentration of 8-isoprostanes in NK cell extracts was measured with the 8-Isoprostane ELISA Kit (Cayman Chemical, Ann Arbor, MI, USA). Samples were provided for ELISA procedure according to the manufacturer’s protocol. Cell lysates were supplied in the presence of 0.005% BHT. Absorbances were measured at 405 nm with Bio-Rad plate reader. A standard curve was prepared with the use of 8-Isoprostane ELISA Standard supplied with the kit and the concentrations of 8-isoprostanes in experimental samples were estimated. Data in the study show the total 8-isoprostane content in NK cell lysates expressed in pg/ml.

### Statistics

All data are expressed as means ± SEM. Normality of data distribution was analyzed by Shapiro-Wilk test. Student’s t test for normal distribution and Mann-Whitney U test for nonparametric distribution were applied to compare two groups. ANOVA test for normal distribution and Kruskal-Wallis test for nonparametric distribution were used to compare the three studied age groups. The multiple comparisons were performed with Tukey’s post-hoc test for normal distribution and Dunn’s post-hoc test for non–parametric distribution. The Spearman correlation coefficient (R) was applied to present the strength of the relationship between variables (Statistica, version 12; Statsoft, Tulsa, OK, USA). Differences or correlations with *p* < 0.05 were considered as statistically significant.

## Results

### Immunological characteristics of the study population

The study population was divided into 3 age groups: ‘young’ (32 subjects, mean age 21 years), ‘old’ (30 seniors at the age under 85, mean age 76 years) and the ‘oldest’ (25 seniors aged over 85, mean age 88 years). Blood morphology of all participants was analyzed prior to the study. The oldest seniors presented normal white blood cell (WBC) count; however, they had more leukocytes in one microliter of peripheral blood than the old ones and this difference was statistically significant (Table 1). All compared groups did not differ significantly in both the number and percentage of lymphocytes. However, seniors under the age of 85 revealed significantly higher percentage of NK cells in the population of lymphocytes than the young subjects, whereas the oldest did not differ significantly from the other age groups. After merging two groups of seniors into one we still observed that the population of seniors presented significantly higher percentage of NK cells compared to the young (seniors: 12.71% vs young: 9.22%; *p* = 0.026; U-Mann-Whitney test).

**Table 1:** Hematological parameters and CRP values of peripheral blood in the studied age groups

Parameters\studied group	Seniors > 85 (oldest), age: 88.4 ± 0.5 y	Seniors < 85 (old), age: 75.6 ± 0.9 y	Young, age: 21.0 ± 0.3 y	*P* value
Total WBC count [k/μl]	6.91 ± 0.34 *	5.92 ± 0.23 *	6.33 ± 0.23	* 0.040
Lymphocyte count [k/μl]	2.17 ± 0.12	2.12 ± 0.12	2.37 ± 0.10	0.246
Lymphocyte percentage [%]	32.22 ± 1.72	36.05 ± 1.87	37.67 ± 1.13	0.059
NK cell percentage [%]	11.06 ± 1.31	14.00 ± 1.49 ^#^	9.22 ± 0.84 ^#^	^#^ 0.034
NK cells CD56^bright^ [%]	4.49 ± 0.78 *	4.15 ± 0.68 ^#^	7.17 ± 0.76 *^#^	^#^ 0.004 *0.037
NK cells CD56^dim^ [%]	93.67 ± 0.71 *	93.58 ± 0.63 ^#^	90.01 ± 0.74 *^#^	^#^ 0.001 *0.002
CRP [mg/L]	2.16 ± 0.29 *	1.87 ± 0.27 ^#^	0.81 ± 0.18 *^#^	^#^ 0.001 *0.00003

All data are presented as means ± SEM. The same symbols ^*, #^ in one row denote statistically significant differences between the marked values

The young people had almost two times more CD56^bright^ cells compared to the oldest and the old ones and significantly less CD56^dim^ cells as compared with the oldest and the old. C reactive protein (CRP) values of all the analyzed groups remained within the reference range (0 to 5 mg/dL), although seniors revealed two times higher values compared to the young (Table 1).

### Expression of SIRT1, SOD2 and HSP70 in non-stimulated NK cells of the elderly and the young

**Fig. 1:**
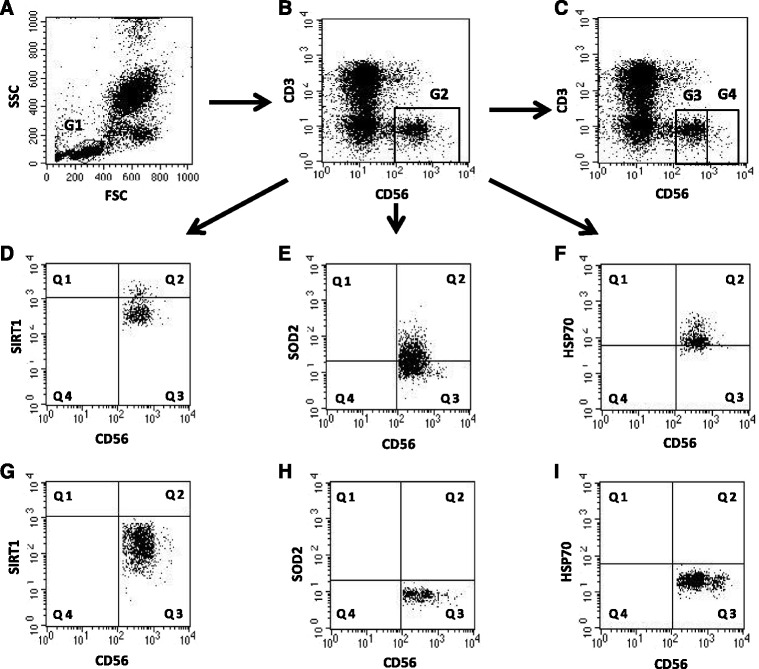
Gating strategy for flow cytometric analyses of NK cells. **a**. Lymphocyte gating - lymphocytes were defined as FSC^low^/SSC^low^ cells (G1). **b**. NK cell gating – NK cells were defined as CD3 negative and CD56 positive population (G2). **c**. NK cell dim/bright gating – NK cells were defined as CD3 negative and CD56 dim positive (G3) or CD56 bright positive population (G4) within NK cell population. **d**. NK cells expressing SIRT1 were identified in upper right quadrant (Q2). **e**. NK cells expressing SOD2 were identified in upper right quadrant (Q2). **f**. NK cells expressing intracellular HSP70 were identified in upper right quadrant (Q2). **g**. Isotype control for SIRT1 positive cells (Q3). **h**. Isotype control for SOD2 positive cells (Q3). **i**. Isotype control for HSP70 positive cells (Q3)

**Fig. 2:**
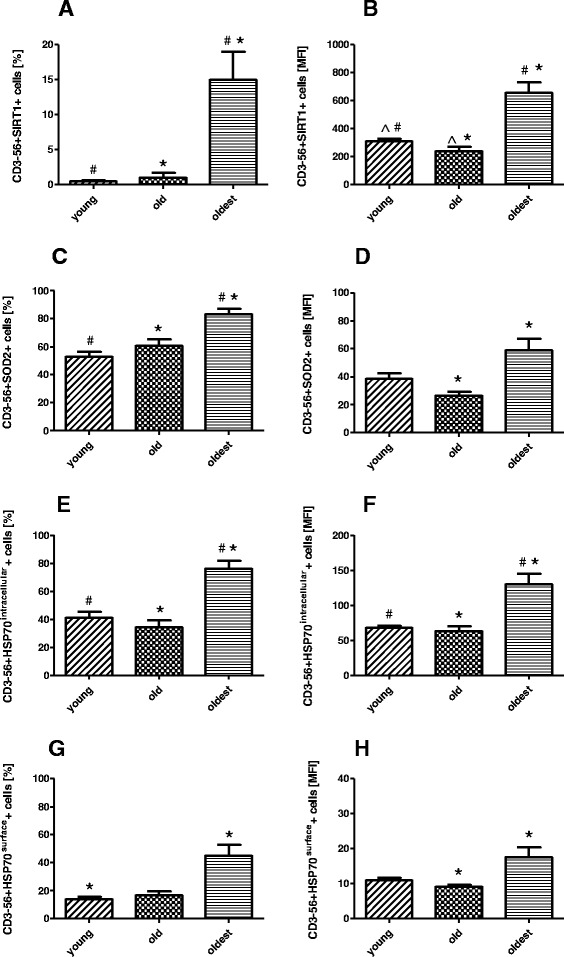
Expression of cellular protective proteins in non-stimulated NK cells of the oldest seniors (aged over 85), the old aged under 85 and the young. Data are presented as means ± SEM and concern expression of the studied proteins in NK cells demonstrated as percentages of cells with expression of a particular protein (%) or mean fluorescence intensity (MFI). The same ^#^,*,^ symbols over bars denote statistically significant differences between NK cells of different age groups. **a**. Expression of SIRT1 (%): ^#^ *p* = 0.0001; * *p* = 0.000002. **b**. Expression of SIRT1 (MFI): ^ *p* = 0.022; ^#^ *p* = 0.004; * *p* = 0.000001. **c**. Expression of SOD2 (%): ^#^ *p* = 0.000009; * *p* = 0.006. **d**. Expression of SOD2 (MFI): * *p* = 0.0003. **e**. Expression of intracellular HSP70 (%): ^#^ *p* = 0.0002; * *p* = 0.000005. **f**. Expression of intracellular HSP70 (MFI): ^#^ *p* = 0.0023; * *p* = 0.000002. **g**. Expression of surface HSP70 (%): * *p* = 0.02. **h**. Expression of surface HSP70 (MFI): * *p* = 0.045

The gating strategy performed for flow cytometric analysis of NK cells is shown in Fig. 1. The expression of SIRT1 was many times higher in NK cells of the oldest seniors (14.99 ± 4.04%) compared to the old and the young (0.99 ± 0.69% and 0.46 ± 0.17%, respectively) (Fig. 2a) and these significant differences were confirmed by measurement of mean fluorescence intensity (MFI) of the analyzed populations (Fig. 2b).

The expression of SOD2 in NK cells of the oldest seniors (83.22 ± 3.92%) differed significantly from NK cells of the old and the young (60.74 ± 4.66% and 52.91 ± 3.38%, respectively) (Fig. 2c) and these results were supported by MFI values of the studied populations, although statistically significant difference was observed only between NK cells of the old and the oldest (Fig. 2d).

The expression of intracellular HSP70 in NK cells of the oldest (76.43 ± 5.47%) was significantly higher than in NK cells of the old and the young (34.34 ± 4.98% and 41.22 ± 4.17% respectively) (Fig. 2e). The similar results were obtained for MFI values of the analyzed populations (Fig. 2f). Differences between age groups in the expression of the surface HSP70 protein revealed statistical significance only between the young and the oldest (Fig. 2g). 45% of NK cells (45.14 ± 7.7%) of the oldest seniors expressed this protein on their surface in contrast to significantly lower expression on NK cells of the young (13.17 ± 1.9%). NK cells of the old also revealed lower expression (16.67 ± 2.98%) but without statistical significance. Results received for MFI values of these populations presented the similar tendency, but the significant difference concerned the old and the oldest (Fig. 2h).

### Correlations between levels of expression of the studied cellular protective proteins in NK cells

It was found that percentages of NK cells expressing SIRT1, SOD2, intracellular HSP70 and surface HSP70 correlated positively with the age of participants (*R* = 0.455, *R* = 0.520, *R* = 0.402 and *R* = 0.320, respectively) (Table 2).

**Table 2:** Correlation analysis of the study population

Spearman’s correlation coefficient (R)	SIRT1	SOD2	HSP70^intracellular^	HSP70^surface^	TNF	IFN-γ	CRP
SOD2	0.517						
HSP70^intracellular^	0.705	0.466					
HSP70^surface^	0.439	0.669	0.418				
TNF	0.262	ns	0.263	ns			
IFN-γ	ns	ns	ns	ns	ns		
CRP	ns	0.274	0.292	ns	−0.262	ns	
Age	0.455	0.520	0.402	0.320	ns	−0.254	0.507

All values are presented as statistically significant Spearman’s correlation coefficients (R). ‘ns’ denotes statistically not significant

Some correlations between the expression of SIRT1 and other protective proteins were observed. The percentage of SIRT1-expressing NK cells correlated with the percentage of NK cells expressing: (i) SOD2 (*R* = 0.517), (ii) intracellular HSP70 (*R* = 0.705), (iii) surface HSP70 (*R* = 0.439) and (iv) TNF (*R* = 0.262) (Table 2).

Moreover, the statistical analysis revealed correlations between the percentage of NK cells expressing SOD2 and the percentage of NK cells expressing: (i) intracellular HSP70 protein (*R* = 0.466), and (ii) surface HSP70 protein (*R* = 0.669). We also noted a correlation between the percentage of NK cells expressing intracellular HSP70 and: (i) surface HSP70 protein (*R* = 0.418), (ii) TNF (*R* = 0.263) (Table 2).

### Correlations between serum concentration of CRP and expression of cellular protective proteins in NK cells

The serum concentration of C reactive protein correlated with age (*R* = 0.507). We found also correlations between CRP and percentages of NK cells expressing SOD2, intracellular HSP70 and TNF (*R* = 0.274, *R* = 0.292 and *R* = -0.262, respectively) (Table 2).

### Intracellular expression of TNF and IFN-γ in non-stimulated NK cells

**Fig. 3:**
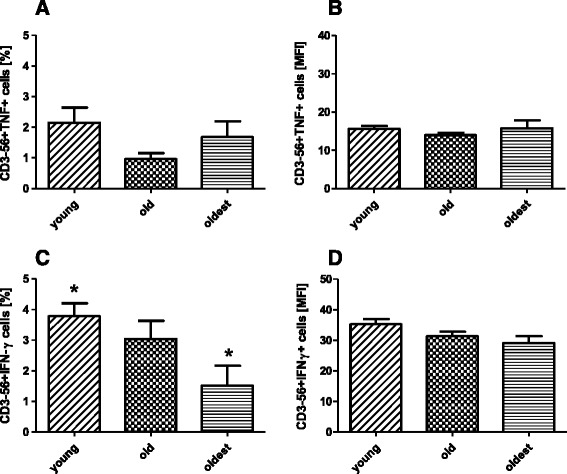
Expression of proinflammatory cytokines in non-stimulated NK cells of the oldest seniors (aged over 85), the old aged under 85 and the young. Data are presented as means ± SEM and concern expression of the studied cytokines in NK cells demonstrated as percentages of cells with expression of a particular cytokine (%) or mean fluorescence intensity (MFI). The same * symbols over bars denote statistically significant differences between NK cells of different age groups. **a**. Expression of TNF (%). **b**. Expression of TNF (MFI). **c**. Expression of IFN-γ (%): * *p* = 0.02. **d**. Expression of IFN-γ (MFI)

Intracellular TNF, a proinflammatory cytokine secreted usually by stimulated NK cells, in our study was detected at a low level also in non-stimulated NK cells of whole blood samples. 2.15 ± 0.49%, 0.97 ± 0.19% and 1.68 ± 0.51% of NK cells of the young, the old and the oldest, respectively, revealed its expression. However, the differences between the studied groups were not statistically significant (Fig. 3a). Similarly, MFI values obtained for the analyzed populations did not show any differences between the age groups (Fig. 3b).

On the contrary, the expression of intracellular IFN-γ in non-stimulated NK cells showed some differences between the age groups. The young had significantly more NK cells expressing intracellular IFN-γ (3.78 ± 0.42%) than the oldest (1.52 ± 0.65%) but they did not differ from the old (3.04 ± 0.59%) (Fig. 3c). The significant differences were not, however, confirmed by MFI values (Fig. 3d).

The percentage of NK cells expressing IFN-γ correlated negatively with age (*R* = -0.254) (Table 2).

### Expression of SIRT1, SOD2 and HSP70 in non-stimulated CD56dim and CD56bright NK cells of the elderly and the young

**Fig. 4:**
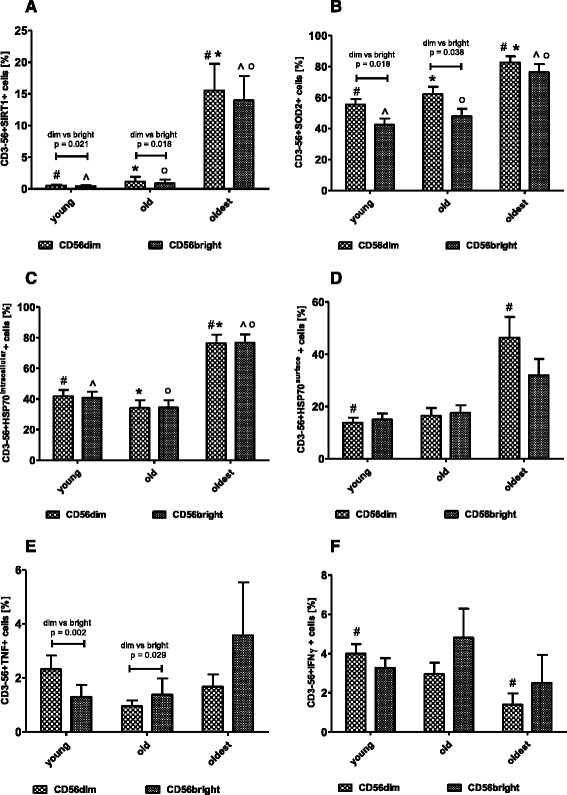
Expression of cellular protective proteins and proinflammatory cytokines in non-stimulated CD56dim and CD56bright NK cells of the oldest seniors (aged over 85), the old aged under 85 and the young. Data are presented as means ± SEM and concern percentages of CD56dim and CD56bright NK cells expressing the studied proteins (%). The same ^#^,*,^,^o^ symbols over bars denote statistically significant differences between respectively CD56dim or CD56bright NK cells of different age groups. Horizontal lines above paired bars denote differences with statistical significance between CD56dim and CD56bright NK cells of the same age group. **a**. Expression of SIRT1 (%): ^#^ *p* = 0.0004; * *p* = 0.000008; ^ *p* = 0.003; ^o^ *p* = 0.002. **b**. Expression of SOD2 (%): ^#^ *p* = 0.00003; * *p* = 0.02; ^ *p* = 0.00007; ^o^ *p* = 0.005. **c**. Expression of intracellular HSP70 (%): ^#^ *p* = 0.0003; * *p* = 0.000005; ^ *p* = 0.0002; ^o^ *p* = 0.000002. **d**. Expression of surface HSP70 (%): ^#^ *p* = 0.02. **e**. Expression of TNF (%). **f**. Expression of IFN-γ (%): ^#^ *p* = 0.01

The gating strategy performed for flow cytometric analysis of CD56dim and CD56bright NK cells is shown in Fig. 1. The expression of SIRT1, SOD2 and both HSP70^intracellular^ and HSP70 ^surface^ was compared between three age groups (Kruskal-Wallis test) and between CD56dim and CD56bright cells of the same age group (Mann-Whitney U test). Interestingly, the general pattern of SIRT1 and HSP70^intracellular^ expression was similar in both CD56dim and CD56bright NK cells (Fig. 4a and c). There were some statistically significant differences between young vs oldest and old vs oldest observed, similarly to the total population of NK cells (Fig. 2a and c). Moreover, the comparison of CD56dim to CD56bright cells revealed also significantly higher expression of SIRT1 in CD56dim cells in both young and the old population but not in the oldest (respectively 0.51 ± 0.18 vs 0.43 ± 0.19 and 1.14 ± 0.76 vs 0.89 ± 0.57). Some differences between CD56dim and CD56bright cells were observed also within SOD2-expressing NK cells. Comparably to SIRT1, in the group of the young and the old, but not in the oldest, CD56dim cells presented significantly higher expression of SOD2 compared to CD56bright cells (55.5 ± 3.48% vs 42.32 ± 4.16% and 62.21 ± 4.73 vs 47.99 ± 4.73, respectively) (Fig. 4b). The expression of HSP70^surface^ (Fig. 4d) was comparable to the expression of SIRT1 and HSP70^intracellular^ with the only significant difference between CD56dim of the young and CD56dim of the oldest observed also in the total population of NK cells (Fig. 2g).

### Intracellular expression of TNF and IFN-γ in non-stimulated CD56dim and CD56bright NK cells

The gating strategy performed for flow cytometric analysis of CD56dim and CD56bright NK cells is shown in Fig. 1. Some differences between CD56dim and CD56bright cells expressing TNF and IFN-γ were observed in the young, old and the oldest. In the young, significantly more CD56dim cells expressed TNF compared with CD56bright cells (2.33 ± 0.5% vs 1.3 ± 0.44%). On the contrary, in the old significantly more CD56bright cells revealed expression of TNF compared with CD56dim cells (respectively: 1.39 ± 0.6% vs 0.96 ± 0.21%). The tendency similar to the old group was observed also in the oldest one but it was not statistically significant (Fig. 4e). Correspondingly, when the expression of IFN-γ was analyzed, some similar to TNF expression trends were observed between CD56dim and CD56bright cells of all three age groups, however, they were not statistically significant. The only significant difference was observed between CD56dim cells of the young vs CD56dim cells of the oldest (Fig. 4f) comparable to the total population of NK cells (Fig. 3c).

### Protein carbonyl groups and 8-isoprostanes content in NK cell lysates

**Fig. 5:**
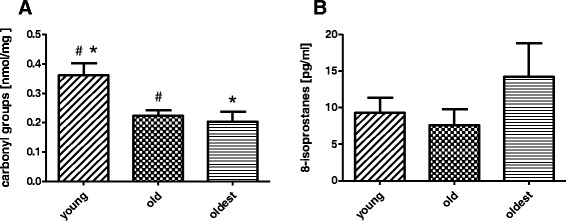
The content of protein carbonyl groups and 8-isoprostanes in NK cell extracts of the oldest (aged over 85), the old aged under 85 and the young. Data are presented as means ± SEM. The same symbols *, ^#^ over bars denote statistically significant differences between age groups. **a** The content of protein carbonyl groups in NK cell extracts expressed as nmol/mg of protein: ^#^ *p* = 0.01; * *p* = 0.02. **b** The concentration of 8-isoprostanes in NK cell extracts expressed as pg/ml

The concentration of carbonyl groups in NK cell extracts of the young (0.36 ± 0.04 nmol/mg of protein) was significantly higher compared to the old (0.22 ± 0.02 nmol/mg) and the oldest (0.21 ± 0.03 nmol/mg) (Fig. 5a). These differences between age groups, however, were not confirmed by changes in concentration of 8-isoprostanes in NK cell extracts of the analyzed populations (Fig. 5b). In these last experiments the highest concentration of 8-isoprostanes was observed in NK cells of the oldest (14.22 ± 4.57 pg/ml) on the contrary to the young (9.26 ± 2.06 pg/ml) and the old (7.59 ± 2.14) but these differences were not statistically significant.

## Discussion

The main finding of this study is that in the oldest seniors NK cells, a specific group of lymphocytes involved in innate immunity, reveal higher expression of stress response proteins, i.e. SIRT1, HSP70 and SOD2 compared to seniors aged under 85 and the young people. The study shows also for the first time the existing correlations between the expression of these proteins in NK cells and age of the study participants.

The studies of immunosenescence provided various data on the number of leukocytes and lymphocytes in peripheral blood. For instance, similarly to our results, no differences between the elderly and the young were reported [4] whereas an Italian study showed a decrease in the number of both leukocytes and lymphocytes in the process of ageing [48]. Although in our study the oldest seniors had significantly more leukocytes per microliter of peripheral blood than the old ones, the values remained within the reference range (4.0 to 10.0 k/μl). Despite similar numbers and percentages of lymphocytes in all compared groups, we found age-dependent alterations in the number of NK cells. Seniors aged under 85 had significantly higher percentage of NK cells in the population of lymphocytes than the young group but after merging the two groups of seniors, the elderly still presented significantly higher percentage of NK cells, similarly to an earlier study by Sansoni et al. who found the increase in the number of NK cells with advancing age [48]. Although another study did not report changes of the absolute NK cell number during ageing, the decrease by 48% in the number of CD56^bright^ NK cells was found in the old compared to the young group [13], similarly to our results. Earlier, Borrego and coworkers found the increase in percentage of NK cells in the elderly due to expansion of CD56dim cells [7]. Thus, immunosenescence is characterized by the expansion of the mature CD56^dim^ subset of NK cells at the cost of immature CD56^bright^ cells.

The expression of SIRT1, SOD2 and HSP70 in NK cells has yet not been investigated during the process of ageing. In our study the oldest seniors presented significantly higher percentage of SIRT1-expressing NK cells compared to the old and the young. Moreover, the positive correlation between the percentage of NK cells with SIRT1 expression and the age of study participants suggests that high expression of SIRT1 may be associated with human longevity. This observation adds up to the increasing evidence that SIRT1 affects lifespan and stress resistance in yeast, worms, flies and mice [6]. Although SIRT1expression was not studied in peripheral blood cells during ageing, a significant increase in SIRT1 concentration in sera of the older people compared to adults and children, and a significant positive correlation between SIRT1 level and age in the overall studied population were observed by Kilic and coworkers [27].

The increased basal expression of SOD2 in NK cells of the oldest seniors compared to the old and the young and a positive correlation between the percentage of SOD2-expressing NK cells and the age of the participants presented in our study underscore the role of SOD2 in healthy ageing of human NK cells. SOD2, similarly to SIRT1, seems to play a role in longevity, at least in some species, i.e. *Drosophila* [43] and mice [46]. However, studies performed on extensive collection of 1,612 long-lived individuals showed no relationships between the analyzed SNPs (Single Nucleotide Polymorphisms) of the SOD2 gene and the longevity phenotype [19].

Our findings of significantly higher expression of intracellular HSP70 protein in non-stimulated NK cells of the oldest seniors and correlation between the expression of this protein and the age of the study participants are in line with the results of a Belgian group. They reported higher levels of HSP70, HSP32 and HSP90, but not HSP27, assessed by flow cytometry in non-stimulated monocytes and lymphocytes of the elderly (mean age 75.0, *n* = 18) compared to young subjects (mean age 36.4, *n* = 17) [41]. These studies, similarly to our observations concerning NK cells, indicate that the increased expression of HSP70, and some other HSP proteins, may be a general feature of leukocyte ageing.

We observed the enhanced expression of HSP70 protein on NK cell surface in the oldest seniors. The membrane association of HSP70 may come, however in two forms, i.e. integrated within the plasma membrane (in tumor cells) or associated with cell surface receptors (in normal cells) [38]. According to these authors commercially available antibodies are able to detect rather receptor-bound HSP70 than the integrated in the plasma membrane. The latter one need specific antibodies that recognize C terminal domain (aa 450-461) of HSP70 molecule [38]. Thus we might observed no expression of HSP70 on the surface of NK cells but rather binding of extracellular HSPs to TLR receptors on the surface of NK cells but more detailed studies are necessary to explain that clearly.

SIRT1 was found to activate several transcription factors of FOXO family that promote the expression of stress response genes including *SOD2* [21]. SIRT1 was also described to control HSF1 (heat shock factor 1) activity, a transcription factor involved in the regulation of expression of chaperons HSP70 and HSP90 [34]. These already known data may indirectly suggest observed in our study correlations between the expression of SIRT1 and SOD2 or SIRT1 and HSP70 in NK cells.

Our studies provided also some interesting observations concerning significantly higher expression of both SIRT1 and SOD2 in CD56dim cells compared with CD56bright cells in the young and the old but not in the oldest. There were no significant differences between CD56dim and CD56bright cells in the expression of both HSP70^intracellular^ and HSP70^surface^ in all age groups. The observed differences seem to be interesting especially regarding the process of ageing. These data are, however, preliminary and need subsequent studies to explain this phenomenon.

The process of ageing is in general characterized by the increased level of oxidative stress [35]. Numerous studies showed a positive correlation between resistance to oxidative stress and maximal lifespan in a variety of mammals, from hamsters to humans [24, 33]. The detected levels of carbonyl groups in NK cells of the studied population, however, did not exceed the normal ranges found in cell lysates, i.e. MRC-5-fibroblasts, 1.3 nmol/mg [51], human plasma, i.e. 1.83 ± 0.4 nmol/mg [1] or serum, i.e. 0.52 ± 0.34 nmol/mg [32]. Thus we did not observe the raise of oxidative stress level in the process of ageing. These data are in line with the results of 8-isoprostane total content test in the analyzed samples. We did not find any significant increase of concentration of 8-isoprostanes in NK cell extracts, which are similarly to carbonyl groups regarded as markers of oxidative stress [36, 37]. Statistically significant differences between carbonyl groups content in NK cells of the young versus old or the oldest were not observed in 8-isoprostane test. Similarly to carbonyl groups, concentrations of isoprostanes in NK cell extracts remained within the normal range found in human plasma and urine (range from 5–40 pg/ml) [37] or breath condensates of healthy subjects (15.8 ± 1.6 pg/ml) [36].

Concentrations of CRP, the acute-phase protein, which level reflects the presence of acute or chronic inflammation, found in the sera of the analyzed subjects, correspond to many data documenting CRP increase with advancing age in apparently healthy humans [5, 42]. Although all participants in our study presented normal CRP values, some age-related differences were observed also within the normal range. It is noteworthy, that in our study CRP serum level correlated positively with the percentage of NK cells expressing cellular protective proteins SOD2 and intracellular HSP70 [10].

The process of ageing is characterized by the increase of serum concentrations of proinflammatory cytokines, i.e. IL-6 and TNF [25, 41]. To test whether non-stimulated NK cells present in the whole blood may contribute to this process the expression of intracellular TNF and IFN-γ, a cytokine considered as a marker of NK activity, was analyzed by flow cytometry. Our study revealed low expression of both cytokines independent on the age of the study participants with the exception of higher expression of IFN-γ in NK cells of the young. In the whole studied population the percentage of NK cells with the expression of IFN-γ correlated positively with the percentage of CD56^bright^ cells (*R* = 0.264) and negatively with CD56dim cells (*R* = -0.321) (data not shown in Table 2). CD56bright cells are the main source of secreted cytokines in NK cells and their number decreases with age [13, 18]. Moreover, the expression of IFN-γ correlated negatively with age. Our results are in line with earlier reports showing a decreased production of IFN-γ in the elderly, although most studies concerned stimulated NK cells [18, 31]. Together with the normal serum levels of CRP, these data suggest that we have studied apparently healthy groups of the young, old and very old individuals.

Analysis of expression of both TNF and IFN-γ in CD56dim and CD56bright non-stimulated NK cells provided also some interesting observations concerning significantly higher expression of TNF in CD56dim cells compared to CD56bright cells in the young. The similar but not statistically significant tendency regarding expression of IFN- γ was also noted. In the old, however, the significantly higher expression of TNF was observed in CD56bright cells compared to CD56dim cells. Then in the oldest the similar, but not statistically significant results were obtained, comparably to IFN-γ expression in both the old and the oldest seniors. Takahashi and coworkers showed that CD56dim cells could be both highly cytotoxic and produce cytokines, similarly to CD56bright cells which could both secrete cytokines and acquire cytolytic activity in some conditions [53]. De Maria and coworkers showed that CD56^dim^ NK cells can produce IFN-γ at 2-4 h after stimulation, but not later and CD56^bright^ cells secrete IFN-γ only at late intervals (over 16 h after stimulation) [15]. Intracellular staining of cytokines was performed within 4 h from blood sample collection so the described by De Maria et al. phenomenon corresponds in our studies to the young group but it does not fit to the old and the oldest. Further studies are necessary to explain these data, especially in the context of ageing process.

The strength of the presented results is based on the careful recruitment of the oldest seniors as they represent healthy mode of ageing and differ significantly from the group of seniors under 85. This advantage counterbalances the limitation of the study, i.e. not large size of the study group (*n* = 87). The other advantage concerns the choice of the analyzed parameters as they have appeared to be involved in the process of NK cell ageing and expand our understanding of immunosenescence and its contribution to longevity.

## Conclusions

Our study provides novel data concerning ageing of the human immune system. The increased expression of cellular protective proteins involved in stress response, i.e. SIRT1, HSP70 and SOD2 in NK cells correlates with the age of study participants and this phenomenon seems to correspond to longevity.

## Abbreviations

ADL: activities of daily living
BHT: butylated hydroxytoluene
CRP: C reactive protein
FBS: fetal bovine serum
FOXO: forkhead transcription factor class O
HSF: heat shock factor
HSP: heat shock protein
IFN: interferon
IL: interleukin
MMSE: mini mental state examination
NK cells: natural killer cells
NSAID: non-steroid anti-inflammatory drug
PBMCs: peripheral blood mononuclear cells
PBS: phosphate-buffered saline
SIRT: sirtuin
SNPs: single nucleotide polymorphisms
SOD: superoxide dismutase
TAC: tetrameric antibody complexes
TLR: toll-like receptor
TNF: tumor necrosis factor
WBC: white blood cell
